# Talking microbes: When gut bacteria interact with diet and host organs

**DOI:** 10.1002/mnfr.201500406

**Published:** 2015-08-26

**Authors:** Patrice D. Cani, Amandine Everard

**Affiliations:** ^1^ Metabolism and Nutrition Research GroupWELBIO‐ Walloon Excellence in Life Sciences and BIOtechnologyLouvain Drug Research InstituteUniversité catholique de LouvainBrusselsBelgium

**Keywords:** Diabetes, Energy metabolism, Gut microbiota, Innate immunity, Obesity

## Abstract

Obesity and diabetes have reached epidemic proportions. Evidence suggests that besides dietary habits and physical activity, other environmental factors, such as gut microbes, are recognized as additional partners implicated in the control of energy homeostasis. Studies on the human gut microbiota have shown that the general population can be stratified on the sole basis of three dominant bacteria (i.e., the concept of enterotypes), while some others have suggested categorizing the population according to their microbiome gene richness. Both aspects have been strengthened by recent studies investigating the impact of nutrients (e.g., dietary fibers, fat feeding) and dietary habits (i.e., vegans versus omnivores) of different populations. Using preclinical models, quite a few novel mechanisms have been proposed in these gut microbiota–host interactions, including the role of novel bacteria, the regulation of antimicrobial peptide production, the maintenance of the gut barrier function and intestinal innate immunity. In this review, we discuss several of the aforementioned aspects. Nonetheless, determining the overall mechanisms by which microbes dialogue with host cells will require further investigations before anticipating the development of next‐generation nutritional interventions using prebiotics, probiotics, synbiotics, or even specific nutrients for promoting health benefits.

**Abbreviations**
COG: Cluster orthologous groups of proteins
GLP‐1: Glucagon‐like peptide‐1
GLP‐2: Glucagon‐like peptide‐2
HFD: high‐fat diet
HGC: high gene count
LGC: low gene count
TLRs: toll‐like receptors
Reg3g: regenerating islet‐derived 3‐gamma

## 1 Introduction

The rising prevalence of obesity is becoming a worldwide concern. The resulting increase in associated metabolic disorders, such as type 2 diabetes, insulin resistance, metabolic inflammation, and nonalcoholic fatty liver diseases, are major risk factors for cardiometabolic disorders and various cancers.

Although the main causes of overweight, obesity, and related disorders reside in inadequate dietary habits and physical inactivity, several other environmental factors are also becoming recognized as important. Over the last 10 years, the intestinal microorganisms (i.e., the gut microbiota) have raised substantial attention. We have known for decades that humans are composed of “only” 10 trillion cells, whereas 100 trillion microbial cells reside in our intestines 1. Thanks to the recent development of analytical tools, we are now enabled to better understand the composition of the human intestinal microbiota and thereby its implication on host physiology 2. The first catalog of 3.3 million nonredundant genes encoded by our gut microbes was published in 2010 3; that catalog has now expanded to 10 million 4. However, besides this important progress achieved thanks to metagenomics analyses, numerous key open questions remain unanswered: *how does gut microbiota develops, how does gut microbiota influences our metabolism? How do dietary factors contribute to shape this microbial community* and *do these key features lead to the onset of metabolic disorders?*

Several recent reviews have discussed the current knowledge on detailed composition of the gut microbiota during obesity, type 2 diabetes, and related diseases. In addition, different mechanisms such as the impact of short‐chain fatty acids, bile acids, or specific metabolites (e.g., TMAO) have extensively been discussed (for review: 5, 6, 7, 8, 9, 10, 11, 12). Moreover, pathobionts such as *Bilophila* have recently been identified 13, 14. The impact of specific probiotic bacteria in the context of metabolic syndrome has been recently reviewed in humans and in rodents 15, 16, Due to these aforementioned reviews, in the present manuscript, we will briefly focus on the history regarding the impact of dietary lipids on gut microbiota composition. Following which, we will also discuss recent data regarding the impact of diet on enterotypes as well as the concept of low gene count (LGC) and high gene count (HGC) in obesity and related diseases. Finally, we will discuss recent data regarding the impact of *Akkermansia muciniphila* on obesity and metabolism, a bacterium particularly interesting in this context.

### 1.1 Dietary lipids modulate the gut microbiota: Focus on rodent studies

In 1965, Graber et al. investigated the impact of high‐fat diet (HFD) feeding on gut microbiota. The authors concluded that “*fecal flora remained relatively stable irrespective of diet*” 17. In 1978, the conclusion of Cummings et al. using the same approach was similar: the authors concluded that “*the fecal microflora including the nuclear dehydrogenating clostridia were unaltered by the dietary changes*” 18. In 1992, Sugawara et al. investigated the impact of HFD ingestion in humans; here again, they concluded that “*no change of fecal flora at the bacterial group level was observed throughout the experimental period, except that the population of lactobacilli showed a tendency to increase in HFD period”* 19. Thus, based on these examples, one would conclude that dietary fatty acids do not influence the composition of intestinal bacteria. However, it is worth noting that all of these studies have investigated the gut microbes using culture‐based approaches. Conversely, using fluorescent *in situ* hybridization and denaturing gradient gel electrophoresis techniques, in 2007, we published that HFD feeding profoundly affected gut microbiota composition in mice 20, 21, 22. Using nonculture‐based approaches, we found that HFD decreases *Eubacterium rectale/Clostridium coccoides*, *Bacteroides* spp., *Bacteroides*‐like MIB, Enterobacteriaceae and *Bifidobacterium* spp. colonization [20, 21]. Interestingly, we found that the decreased abundance of *Bifidobacterium* spp. was inversely correlated with body weight, fat mass, insulin resistance, and low‐grade inflammation 21. Therefore, these findings were not in accordance with previous studies using culture‐dependent approaches. In 2008, Turnbaugh et al. confirmed and extended our findings using pyrosequencing methods and clearly showed that HFD feeding affects both gut microbiota composition and related metabolic functions 23. These findings strongly suggested that preliminary earlier studies investigating gut microbiota composition on host metabolism should be taken with caution and should be eventually reconsidered with novel approaches. One of the limitations of these observations is that the majority of the experiments have been obtained in rodent studies. In addition, recent evidence suggests that the results may be highly dependent on housing conditions or sources of rodent diets 24, 25, 26, 27. However, it is worth mentioning that besides these limitations, numerous other studies have confirmed that dietary fatty acids are major components of the diet influencing the gut microbiota composition, nevertheless the impact on specific taxa is not always consistent 28, 29, 30, 31, 32, 33, 34, 35, 36, 37, 38, 39.

### 1.2 Diet, microbial diversity, and metabolic disorders: Focus on human studies

A recent paper by Doré and Blottière reviewed the impact of diet on gut microbiota with a specific focus on microbial diversity (i.e., species richness of the microbiota) in human studies (for review 5). Notably, several publications suggest that lifestyle and diet not only influenced the composition of the gut microbiota but also impacted metabolic functions. Low bacterial richness consistently appears as a risk factor for different diseases (e.g., intestinal inflammation, obesity, insulin resistance, low‐grade inflammation) 40. In addition to the concept of clustering subjects on the basis of microbial diversity, in 2011, Arumugam et al. introduced the so‐called enterotypes concept. They showed that the human population could be stratified on the simple basis of three microbial patterns dominated by *Bacteroides*, *Prevotella* or, to a lower extent, by *Ruminococcus* 41. The same year, Wu and colleagues reported that long‐term dietary habits were associated with the dominance of one specific genus belonging to these enterotypes 42. Interestingly, they observed that subjects ingesting a diet particularly rich in protein and animal fat were associated with the *Bacteroides* enterotype, whereas subjects ingesting more carbohydrates were dominated by *Prevotella* 42. In a controlled‐feeding study involving ten subjects, Wu and colleagues showed that gut microbial changes occurred within 24 h of initiating a high‐fat/low‐fiber or a low‐fat/high‐fiber diet, though without affecting enterotypes. Recently, David et al. extended this finding by investigating the impact of the short‐term consumption of diets fully composed of animal or plant items. They found that dietary habits altered the microbial community in a nutrient source‐dependent manner within 24 h 43. The concept of enterotypes has been questioned 44, however several data suggest that although well existing, the enterotypes are probably not always strictly restricted to three genera 45, 46. The stability of the enterotypes over time has been recently validated in a cohort of Korean monozygotic twins. During longitudinal analysis, Lim and colleagues found that healthy Koreans were clustered into *Bacteroides* and *Prevotella* enterotypes 47. They found that 13/18 of the monozygotic twins (72.2%) shared the same enterotype. Remarkably, after an average of 2 years, only 3/16 subjects (18.7%) showed enterotype changes, thereby suggesting that the enterotype of each subject remained stable over time 47. This study is in line with the previous reports showing that neither 10 days nor 6 months of dietary changes were sufficient to switch enterotypes 42, 48. Strikingly, besides these two observations regarding either enterotypes or the overall intestinal microbiota, Wu et al. recently studied the effect of diet on the gut microbiota and the host metabolome in healthy vegans and omnivores living in urban environments 49. Interestingly, although the gut microbiota were modestly affected by the participants dietary habits (i.e., vegans versus omnivores), 25% of the plasma metabolites differed between groups. They also found that the vegan metabolomes were enriched in metabolites originating from the gut microbiota. In contrast with the diet, no unique bacteria were significantly correlated with metabolites 49.

Although enterotypes are not associated with body mass index, age, sex, or ethnicity, recent evidence suggests that subjects may be classified on the basis of the number of bacterial genes (i.e., microbial gene richness). Using metagenomic approaches, Le Chatelier et al. identified a bimodal distribution of microbial genes leading to the stratification of the population as either LGC or HGC according to the number of genes harbored 40. Using distinct cohorts of individuals, they showed that microbial gene richness and, eventually, gut microbiota composition were strongly associated with host metabolic markers, such as body weight, fat mass, inflammation, glucose, and lipid metabolism 40. The authors also found that obese LGC individuals gained more weight over time and experienced increased adiposity, insulin resistance, and inflammation compared with HGC individuals 40. Interestingly, they also showed that microbial gene richness could be modified in part by dietary intervention. In fact, Cotillard et al. demonstrated that energy restriction, including different dietary fibers, may increase diversity up to 25%, but this increase was observed only in LGC subjects 50. More fascinatingly, they found that dietary restriction in overweight or obese patients was less efficient in LGC than in HGC individuals in terms of body weight loss, improvement of insulin sensitivity, and decrease of inflammation. These observations strongly suggest that subjects are not equal in terms of responsiveness to dietary intervention and, eventually, body weight loss upon calorie restriction. This hypothesis implies, from a conceptual point of view, that gut microbiota composition and gene richness may be used as predictive factors to stratify the putative potential efficacy of dietary intervention 50.

### 1.3 Gut microbiota modulation and specific host responses

Assuming that the gut microbiota composition and its metabolic capacity directly contribute to regulate host metabolism is widely accepted 26, 51, 52; however, the mechanisms involved in these complex cross‐talk events are only beginning to emerge. Herein, we will limit our discussion to mechanisms discovered in preclinical studies and involving the onset of low‐grade inflammation and metabolic disorders associated with obesity.

**Figure 1:**
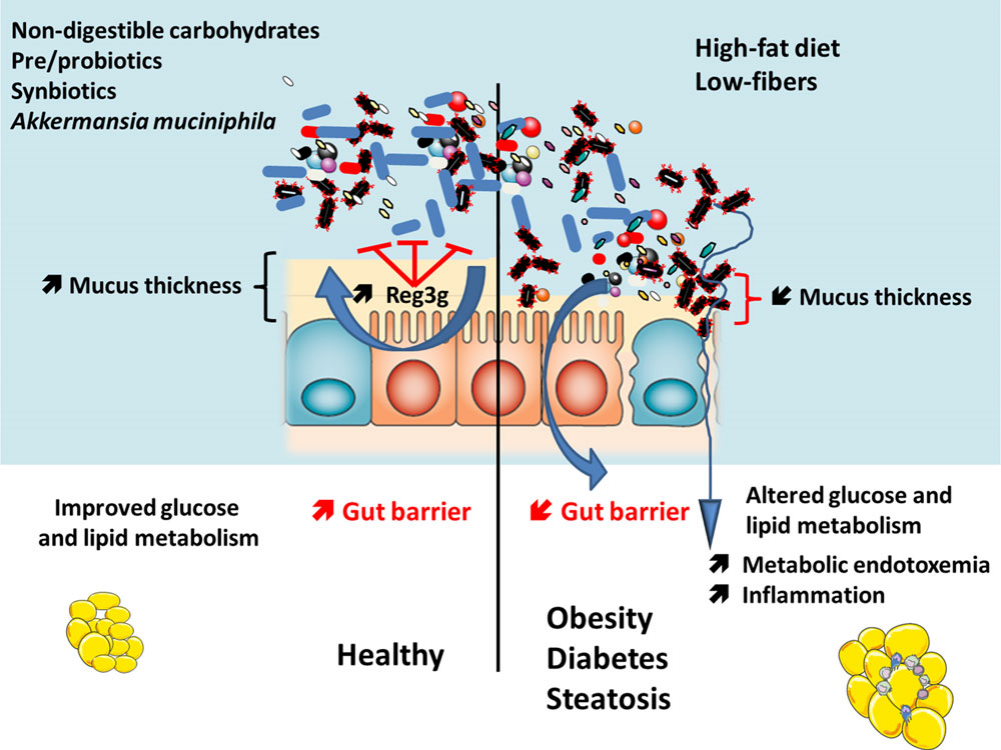
Crosstalks between host and microbes: impact on metabolism. The intestinal barrier is composed of different factors such as epithelial cells, a mucus layer, and antimicrobial peptides produced by host cells. The inner mucus layer and the antimicrobial peptides help to segregate microbes at distance of the epithelium. Moreover, specific microbes such as *Akkermansia muciniphila* have been shown to improve gut barrier function and mucus layer thickness. During high‐fat diet feeding and low fibers intake, the gut microbiota composition is different, inflammatory components translocate into the blood via the altered gut barrier function.

We have previously demonstrated that specific products of intestinal bacteria are involved in the pathophysiology of insulin resistance and type 2 diabetes 20, 21. In 2007, we found that changes in gut microbiota composition observed following HFD feeding were associated with increased plasma lipopolysaccharide (LPS) levels (the so‐called metabolic endotoxemia) 20, a phenomenon also extensively confirmed in other studies and in humans (Fig. 1) 29, 53, 54, 55, 56, 57, 58, 59, 60, 61, 62, 63, except in a recent study 64. The reason of this discrepancy is likely due to the difficulty to asses these critical parameters in complex matrix such as blood (e.g., false negative and LPS recoveries). Because LPS is a constituent of Gram‐negative bacteria, this change indicates a potential implication of the gut microbiota. We gradually discovered different mechanisms explaining the origin of this metabolic endotoxemia during obesity and diabetes. For example, we discovered that dietary fatty acids increase gut permeability, thereby leading to the translocation of intestinal luminal factors 22, 65. The gut barrier function is a complex system involving numerous factors, such as physical factors (e.g., mucus layer thickness, mucus composition, and tight junctions), innate immunity factors (e.g., toll‐like receptors (TLRs), antimicrobial compounds, and intestinal epithelial infiltrated immune cells) or even specific microbes playing putative beneficial roles (e.g., *Bifidobacterium* spp., *A. muciniphila*, and *Faecalibacterium prausnitzii*) (Fig. 1). Over the last few years, preclinical studies helped us to identify different pathways potentially involved in the development of metabolic disorders associated with obesity. Using nondigestible food ingredients to stimulate growth and/or activity of gut bacteria in ways claimed to be beneficial to health, such as prebiotics, several novel mechanisms of interactions between bacteria and host have been studied (for review 66, 67, 68, 69). Previous studies using culture‐independent methods (i.e., %G+C profiling method) have shown that prebiotics affect gut microbiota composition 70. More recently, using deep metagenomic sequencing we found that prebiotic feeding not only affects gut microbiota at the taxonomic level but also profoundly changes metabolic functions of the gut microbiota during both normal diet and HFD feeding 35. A total of 20 genera were significantly affected by the HFD compared to the control diet, whereas prebiotic treatment mitigated the impact of HFD on gut microbiota composition and metabolic functions, along with host metabolic parameters, such as obesity, diabetes, and inflammation 35.

Importantly, we also found that microbiomes from mice fed different diets (control diet, prebiotic, or HFD) showed similar distributions of the occurrence of clusters of orthologous groups of proteins (COG) categories (i.e., classes). However, for 85% of the COG classes, we detected significant modifications in their abundance between the normal diet and at least one of the three other diets, such as for instance changes in genes involved in amino acid transport and metabolism, in lipid metabolism, in energy production and conversion, in cell motility, in nucleotide transport and metabolism or inorganic ion transport and metabolism 35. In accordance with these data, another study also reports that high‐fat diet feeding alters the biochemical composition of the gut microbiota either by changing phylotype composition, metabolic signatures at different levels such as pathways of amino acid metabolism, energy production and conversion, but also lipid metabolism 39. Together, these data demonstrate that the dietary interventions change gut microbiota richness and diversity at both the functional and taxonomic levels, thus providing a basis for the detection of novel interesting taxa or metabolic functions that are potentially involved in the development of metabolic disorders induced by HFD feeding.

Together, this work, combined with other studies, led us to identify novel pathways by which gut microbes interact with host cells and vice versa, hence leading to changes in gut barrier and metabolic inflammation.

For example, in 2011, we demonstrated that prebiotic feeding increases the abundance of *A. muciniphila*, a novel mucin‐degrading bacteria living in the mucus layer (Fig. 1) 71. This bacterium vastly colonizes the mucus layer localized at the host epithelial interface in the ileum and the colon 72. We found that the higher levels of *A. muciniphila* were positively correlated with the number of enteroendocrine L cells producing glucagon‐like peptides 1 and 2 (GLP‐1 and GLP‐2), two peptides involved in energy and glucose metabolism and gut barrier function, respectively 73. Two years later, we demonstrated that feeding mice with *A. muciniphila* reduces body weight gain, fat mass development, and low‐grade inflammation and restores gut barrier function by both acting on mucus layer thickness and restoring the production of specific antimicrobial proteins (i.e., regenerating islet‐derived 3‐gamma [Reg3g]) 74. Interestingly, the latter effect is also observed following prebiotic treatment 35 (Fig. 1). The impact of *A. muciniphila* on glucose metabolism and gut barrier function has been recently confirmed 75. Strikingly, a recent study has shown an increased abundance of *A. muciniphila* following high‐fat and high carbohydrate (sucrose, maltodextrin, corn starch) diet feeding, but was not associated with any metabolic markers 37. In addition, whether one part of the carbohydrates contained in the high‐fat diet were nondigestible, thus promoting the bloom of *A. muciniphila* as previously observed 35, 73, 76, 77, remains to be investigated. Conversely, Chaplin et al. did not find specific impact of high‐fat diet feeding on the abundance of *A. muciniphila*, however, they found that feeding mice with a high‐fat diet‐enriched with conjugated linoleic acid increases intestinal *A. muciniphila* levels 38.

Together, these findings strongly suggest that particular gut microbes such as *A. muciniphila* contribute to the maintenance of the gut barrier through several complementary mechanisms including the production of short chain fatty acids and the regulation of transcription factor or genes controlling cell cycle, lipolysis, and satiety as recently described by Apajalahti et al. 78. Reunanen et al. extended our previous findings *in vivo* 74 by showing *in vitro* (i.e., Caco‐2 and HT‐29 human colonic cell lines models) that *A. muciniphila* adheres to the intestinal epithelium and strengthens the gut barrier by interacting with the host mucosa, but not to human colonic mucus 79.

Although, several beneficial associations have been found between *A. muciniphila* and metabolism, other reports have found among other bacteria a higher abundance of *A. muciniphila* in models of colon cancer 80. Fasting and starving situations are associated with higher abundance of this bacterium 81, 82, thus whether this association is involved in the etiology of such diseases or is the consequence of a change in dietary habits upon such pathologies required further investigations.

Nevertheless, in accordance with the data obtained in rodents, we recently show in obese humans, that in the basal state, the abundance of *A. muciniphila* is inversely related to fasting plasma glucose levels, visceral fat accumulation, and adipocyte diameter in subcutaneous adipose tissue 83. More precisely, subjects with higher gene richness (HGC) and *A. muciniphila* abundance have a lower fasting glucose, triglycerides, and lower body composition. In addition, upon caloric restriction, obese individuals with higher baseline *A. muciniphila* displayed greater improved insulin sensitivity markers and other cardiometabolic risk factors 83. Thus, whether specific interventions, such as nutrients increasing the intestinal levels of *A. muciniphila* or its administration, are of interest and merit further investigation in humans.

The tools summarized above support the notion that changing the gut microbiota may impact host metabolic inflammation, likely by acting on gut barrier function. However, one would argue that discovering interactions between specific bacteria or dietary factors and host metabolism is rather limited to one part of the puzzle. For instance, from the host point of view, the mechanisms leading to the increased inflammation, fat accumulation, insulin resistance, liver steatosis, and gut barrier dysfunction following fat consumption are not fully understood. Thus, the specific relationships with the gut microbiota involved in the onset of these diseases are still a matter of debate.

### 1.4 Dialogues between host and microbes control energy homeostasis

Intestinal epithelium is the first organ in contact with food and nutrients. This organ is also considered to be the largest surface of exchange with both the exterior and gut bacteria. Although the roles of the vast majority of TLRs are known (e.g., specific recognition of pathogens, such as bacteria, viruses, or components of such microorganisms), numerous studies have investigated the impact of knocking down one or another of the TLRs in an entire organism in the context of obesity and type 2 diabetes 20, 22, 25, 84, 85, 86, 87, 88, 89, 90. However, the intestinal innate immune system is likely one of the greatest factors involved in the interactions between gut microbes and the host; therefore, the organ (i.e., intestine) specificity of such communications is also essential.

MyD88 (myeloid differentiation primary response gene 88) is a central adaptor molecule of most of the TLRs. Thus, given that the MyD88 protein is at the interface of the interaction between microorganisms and the host, it may be viewed as a central switch in these multifaceted cross‐talk events. With this possibility in mind, we recently examined the hypothesis that MyD88 in the intestinal epithelial cells acts as a sensor involved in the interaction between nutrients, gut microbes and the host during diet‐induced obesity 36. For this purpose, we generated a mouse model with an inducible intestinal epithelial specific deletion of MyD88. The power of this model is that it allows normal development of the immune system and circumvents putative adaptation of the immune system and the gut bacteria during development. Indeed, with this inducible model, we were able to induce MyD88 deletion in adult mice. We found that under a normal control diet, deleting MyD88 in the intestinal epithelial cells did not alter host metabolism in terms of body weight, food intake, glucose tolerance, inflammatory tone, and fat mass development. However, we did detect an increased signature of regulatory T (Treg) cells in the intestinal epithelium 36. Strikingly, when the mice were switched onto an HFD, they were partially resistant to diet‐induced obesity, fat mass development, and insulin resistance. This finding strongly suggests that fat feeding requires, at least in part, a signal coming from the host intestinal cells to induce obesity and metabolic disorders. When analyzing the specific impact of the deletion, we discovered that intestinal epithelial cells in MyD88‐deleted mice exhibited significantly higher energy expenditure than the obese and diabetic mice, without affecting energy intake (Fig. 1) 36. Thus, these data suggest that intestinal epithelial MyD88 is a sensor changing host metabolism according to the diet, thereby influencing energy metabolism. Interestingly, it is likely that this protection occurs through the reinforcement of the gut barrier at different levels, as we detected an increase in antimicrobial peptide production (e.g., Reg3g), a higher abundance of intestinal epithelial Treg cells and increased levels of markers of anti‐inflammatory molecules and intestinal cell proliferation (i.e., IL‐18 and endocannabinoids) in intestinal epithelial cell MyD88‐deleted mice under an HFD compared to wild‐type mice under an HFD.

Along the same line, a recent study by Chassaing et al. shows that the ingestion of dietary emulsifiers (i.e., carboxymethylcellulose and polysorbate 80) dramatically altered the gut lining. They found that chronic ingestion of such compounds reduces the mucus layer thickness and was involved in the onset of intestinal inflammation, obesity, and diabetes. These effects were associated with an increased food intake, from unknown origin. Interestingly and in accordance with previous studies described in this review, this study highlights putative links between the mucus layer (i.e., decreased thickness), gut microbes, and host metabolism 91.

Although these studies are encouraging, numerous further works are necessary to delineate the specific mechanisms contributing to this phenotype. For instance, *what are the bacteria and/or the metabolites increasing energy expenditure? Are any host metabolites involved in this effect?*

## 2 Conclusion

Altogether, the current literature provides evidence that symbiotic communications exist between not only gut microbes and the host but also between the host and microbes. These cross‐talk events are regulated by fine‐tuned mechanisms leading to the tolerance of commensals and by selecting presumed beneficial microbes.

Both dietary habits and intrinsic host parameters (e.g., genetic, immunity) directly contributed to shape the gut microbiota. However, while some factors appear to be firmly stable, such as enterotypes, some others are more susceptible to change within hours, such as microbial signatures (i.e., metabolite production or specific taxa). Finally, recent preclinical interventions have demonstrated that intestinal epithelial cells and immunity contribute to switch metabolic status according to the ingested nutrients.

Thus, while it is nearly impossible to provide a holistic view of the dialogue existing between us and our gut inhabitants at this stage of current knowledge, we still have the option to adjust our dietary habits toward specific foods or nutrients susceptible to modulating the gut microbiota (e.g., prebiotics, probiotics, polyphenols 15, 16, 77) to improve dietary patterns and health outcomes.

*The authors have declared no conflict of interest*.
